# Scalable Production of AAV Vectors in Orbitally Shaken HEK293 Cells

**DOI:** 10.1016/j.omtm.2018.11.004

**Published:** 2018-11-22

**Authors:** Daniel Blessing, Gabriel Vachey, Catherine Pythoud, Maria Rey, Vivianne Padrun, Florian M. Wurm, Bernard L. Schneider, Nicole Déglon

**Affiliations:** 1 Department of Clinical Neurosciences, Laboratory of Neurotherapies and Neuromodulation (LNTM), Lausanne University Hospital (CHUV), 1011 Lausanne, Switzerland; 2 Neurosciences Research Center (CRN), Laboratory of Neurotherapies and Neuromodulation (LNTM), Lausanne University Hospital, 1011 Lausanne, Switzerland; 3 Brain Mind Institute, Ecole Polytechnique Fédérale de Lausanne (EPFL), 1015 Lausanne, Switzerland; 4 ExcellGene SA, 1870 Monthey, Switzerland; 5 Faculty of Life Science, Ecole Polytechnique Fédérale de Lausanne (EFPL), 1015 Lausanne, Switzerland

**Keywords:** adeno-associated virus, AAV8, AAV9, suspension HEK293 cells, transient transfection, orbitally shaken bioreactors, immune affinity chromatography, CNS transduction

## Abstract

Adeno-associated virus (AAV) vectors are currently among the most commonly applied for *in vivo* gene therapy approaches. The evaluation of vectors during clinical development requires the production of considerable amounts of highly pure and potent vectors. Here, we set up a scalable process for AAV production, using orbitally shaken bioreactors and a fully characterized suspension-adapted cell line, HEKExpress. We conducted a proof-of-concept production of AAV2/8 and AAV2/9 vectors using HEKExpress cells. Furthermore, we compared the production of AAV2/9 vectors using this suspension cell line to classical protocols based on adherent HEK293 cells to demonstrate bioequivalence *in vitro* and *in vivo*. Following upstream processing, we purified vectors via gradient centrifugation and immunoaffinity chromatography. The *in vitro* characterization revealed differences due to the purification method, as well as the transfection protocol and the corresponding HEK293 cell line. The purification method and cell line used also affected *in vivo* transduction efficiency after bilateral injection of AAV2/9 vectors expressing a GFP reporter fused with a nuclear localization signal (AAV2/9-CBA-nlsGFP) into the striatum of adult mice. These results show that AAV vectors deriving from suspension HEKExpress cells are bioequivalent and may exhibit higher potency than vectors produced with adherent HEK293 cells.

## Introduction

Viral vectors have a long track record in therapeutic gene delivery and intervention. Current efforts are starting to bear fruit, as more viral vector therapies are reaching the later stages of clinical development. Glybera, an adeno-associated virus serotype 1 (AAV1)-based gene therapy approach to treat lipoprotein lipase deficiency, was the first viral vector therapeutic approved by the European Medicines Agency (EMA) (in 2012).^1^, ^2^ Recently, the US Food and Drug Administration (FDA) has approved Spark Therapeutic’s Luxturna, another AAV-based gene therapy, to treat an inherited condition that leads to blindness. All viral vector therapeutics that received “Priority Medicine” status by the EMA in 2017 are recombinant adeno-associated virus (rAAV) vectors, reflecting the popularity of AAV vector technology and its therapeutic potential.^3^

Discovered in 1965, AAVs are non-pathogenic members of the *Dependovirus* genus of parvoviruses, endemic in humans.^4^, ^5^ Productive infection requires co-infection with a helper virus, such as adenovirus or herpes virus. Since the discovery of AAV, a variety of serotypes and variants have been described and characterized, with AAV2 being the most extensively studied. The single-stranded DNA (ssDNA) genome, which is flanked by two inverted terminal repeats (ITRs), can be replaced by any gene (maximum ≈5kb) to create a rAAV vector genome.^6^ AAV vector technology has advantages that make it one of the most attractive solutions for therapeutic gene delivery. It is possible to transduce both dividing and non-dividing cells with AAV vectors, and long-term transgene expression can be achieved in post-mitotic cells. Furthermore, AAV exhibits low immunogenicity, and no adverse events have been reported during past clinical trials.^7^

Hurdles and limitations have become apparent as the technology has matured and product development has intensified, prohibiting the full translation of basic research to the clinic and the market. Testing a therapeutic candidate in clinical trials poses a serious challenge concerning scale-up. A process that allows the robust and reproducible manufacturing of a drug at the required scale, with the required yields and purity, is key for its clinical development and commercial viability.

Despite the availability of scalable methods and protocols for production, transient transfection of adherent HEK293 cells remains the most commonly used method to produce AAV vectors for pre-clinical research. The transfection of Sf9 cells, using the baculovirus expression vector system (BEVS), allows large-scale production with high-volumetric yields.^8^, ^9^ However, this expression system is less frequently used in basic research. Recently, several groups have demonstrated the technical feasibility of scaling up AAV production by transfecting suspension-adapted HEK293 cells.^10^, ^11^

Here, we report the implementation of a scalable process for the production of AAV vectors using suspension HEKExpress cells in orbitally shaken bioreactors (OSRs) and polyethylenimine (PEI)-mediated transient transfection. Key features of OSR technology are high gas transfer rates, low mixing times, and low specific power consumption.^12^, ^13^, ^14^ OSRs can be operated on a scale from 5 mL to 1,000 L and have shown excellent scalability.^15^ We produced AAV2/8 and AAV2/9 vectors in suspension using orbital shaken bioreactors. Additionally, we conducted a side-by-side comparison of AAV2/9 production in adherent and suspension-adapted HEK293 cells to validate and demonstrate bioequivalence. The adherent cells were transfected following an established protocol for calcium phosphate transfection.

The purpose of this study was to validate a newly implemented process that offers scalability, compliance, and economic advantages. We demonstrated the potency of vectors produced using suspension-adapted HEK293 cells by comparing them with vectors produced in classical adherent HEK293 cell cultures. We assessed bioequivalence by analyzing the vectors produced by the two methods, both *in vitro* (immunoblot, electron microscopy [EM], ELISA) and *in vivo*, to determine transduction efficiency in the CNS.

## Results

### Cultivation and Transfection of HEK293 Cells in Suspension

We selected the HEKExpress cell line, which has been previously adapted to growth in suspension and characterized according to good manufacturing practice (GMP) guidelines by the provider. We assessed the capacity of HEKExpress cells to be maintained in serum-free F17 Freestyle medium using OSRs for scalable production. The advantage of the OSR system for small and larger scale cell culture under suspension conditions has been verified in direct comparisons.^12^, ^13^, ^15^, ^16^, ^17^ OSR technology has been validated by its widespread use in industry and is used at small scale (10–30 mL scale) by most of the pharmaceutical companies involved in protein expression, particularly for process development and prediction of the theoretical yield when scaling processes up, even if scale-up is performed in stirred tank bioreactors. We implemented the OSR platform for AAV production by initially recording the growth profiles of the HEKExpress cells in Freestyle F17 medium, principally cell density and viability (Figure S1). Small-scale transfections were carried out using a protocol previously described by Chahal et al.^10^ We used 25-kDa linear PEI as a transfection reagent. PEI with these specifications can be obtained in GMP quality. For vector production, we applied a two-plasmid system, including a helper plasmid (pDP8 or pDP9) and a shuttle plasmid (AM/CBA-EGFP-WPRE-bGH or AAV-cytomegalovirus (CMV)-GFP). For transfections, PEI and plasmid DNA were mixed and incubated in F17 medium before being added to a diluted cell culture (1 × 10^6^ cells/mL). The possibility to dilute the cell culture and perform the transfection in the presence of conditioned medium, without requiring medium exchange, is a key feature for a scalable vector manufacturing process. We observed high viability throughout the exponential growth of the cells, as well as high transfection efficiencies and rAAV synthesis (Figure S1). The amount of produced intracellular vector peaked at 72 hr, in accordance with previous studies.^18^

### Production and Purification of rAAV Vectors for a Comparative Study

**Figure 1:**
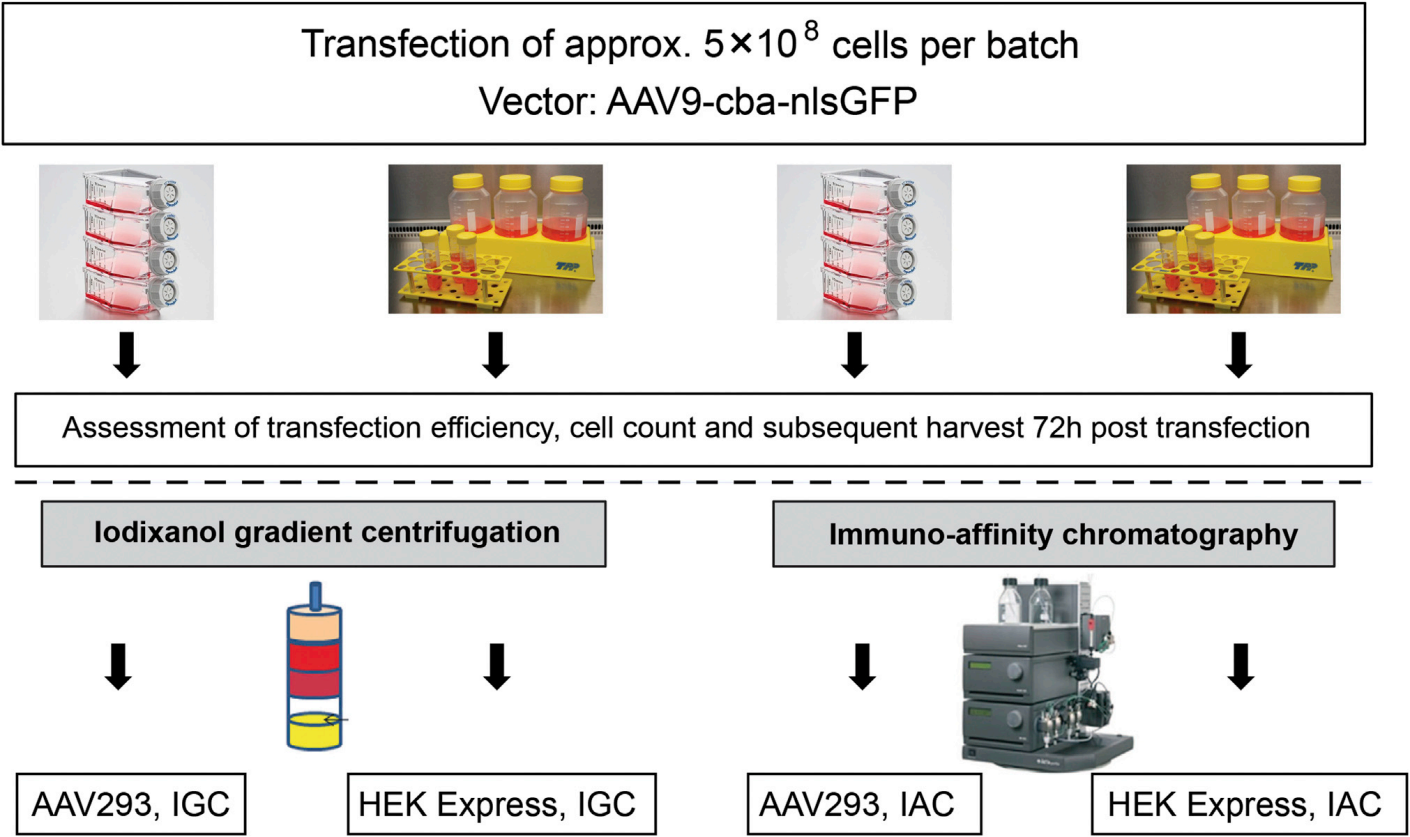
Scheme for the Side-by-Side Comparison of Production Methods and Purification Procedures HEK293 cells were transfected using calcium phosphate transfection for adherent AAV293 cells grown in cell factories and PEI for HEKExpress suspension cells grown in Tubespin 600 bioreactors. A total of 5 × 10^8^ cells (500 mL) per batch were transfected and harvested 72 hr post-transfection. Lysates were purified via gradient ultracentrifugation (IGC) or immunoaffinity chromatography (IAC). Four vector preparations (AAV293 IGC, HEKExpress IGC, AAV293 IAC, and HEKExpress IAC) were generated for this study.

The main objective of this study was to investigate the production of AAV vectors in suspension using OSR. Furthermore, we aimed at comparing the yield and potency of vectors produced using PEI-mediated transfection of HEKExpress cell suspensions with that using calcium phosphate-mediated transfection of adherent AAV293 cells (Figure 1). We produced AAV2/8, expressing a GFP reporter (AAV2/8-CMV-GFP). Following this first feasibility study, we used the same protocol to produce AAV2/9, expressing a GFP reporter fused with a nuclear localization signal (AAV2/9-CBA-nlsGFP). The AAV8 and AAV9 serotypes were chosen because they are potent vectors for CNS gene delivery.^19^, ^20^, ^21^ We produced these vectors at a scale of 500 mL (n = 2) using orbital shaken cell suspensions and harvested the cells 72 hr post-transfection. The AAV2/9 vector was in parallel produced with adherent cells using a classical protocol for side-by-side comparison. The transfection efficiencies of adherent cells using calcium phosphate (83%–86%) were higher as compared with suspension cells and PEI-mediated transfection (60%–61%) (Table 1). However, the number of cells in suspension (1 × 10^9^ cells per batch) was 2-fold higher than the number of adherent cells 72 hr post-transfection. Thus, the total number of cells expressing the nuclear GFP transgene was higher for the transfected cells in suspension.

**Table 1:** Summary of Parameters for Batch Production Using Adherent and Suspension-Adapted HEK293 Cells

Cell Line	AAV293 (Adherent)		HEKExpress (Suspension)	
	AAV293 1 (IGC)	AAV293 2 (IAC)	HEKExpress (IGC)	HEKExpress (IAC)
Bioreactors per batch	5 cell factories (500 cm^2^)	5 cell factories (500 cm^2^)	2 TubeSpin 600	2 TubeSpin 600
Medium	500 mL DMEM (+FBS)	500 mL DMEM (+FBS)	500 mL F17	500 mL F17
Cell number at transfection	≈500 × 10^6^^a^	≈500 × 10^6^^a^	500 × 10^6^	500 × 10^6^
Cell number at harvest	456 × 10^6^	480 × 10^6^	1 × 10^9^	1.1 × 10^9^
Plasmid DNA per batch	1.5 mg	1.5 mg	0.75 mg	0.75 mg
Transfection method	calcium phosphate	calcium phosphate	PEI	PEI
Transfection efficiency	83%^b^	86%^b^	60%	61%

AAV293 cells were seeded with 20 × 10^6^ cells per cell factory (100 cm^2^). HEKExpress cells were seeded at 1 × 10^6^ cells/mL. All cultures were incubated at 37°C for 72 hr. Cells in suspension were incubated with a shaking frequency of 180 rpm. Transfection efficiencies for adherent cell batches were determined by manual counting of GFP-positive cells, whereas that of cells in suspension was measured by flow cytometry.

a Approximate cell number calculated based on the confluence of adherent cultures.

b Determined by manual counting using a fluorescence microscope and hemocytometer.

In addition to the comparison of production methods, we also compared the purification of vectors via iodixanol gradient centrifugation (IGC) and immunoaffinity chromatography (IAC) (Figure 1). Gradient centrifugation purification is commonly used for lab-scale and cell-pellet harvesting.^22^ This approach is not suitable for studies in large-animal models or clinical trials, because it would be very laborious to process larger liquid volumes. We thus tested purification by chromatographic capture via affinity ligands, which is scalable and compliant. We chose the POROS CaptureSelect AAV8 and AAV9 resins for IAC as single-step purification. Chromatographic capture with this resin significantly reduced impurities in a single process step. The nature of the resin and its mechanical resistance to pressures up to 10 MPa allows the application of high linear velocities. Hence it is ideally suitable for processing large liquid volumes in a manufacturing scale.

### Titration of AAV2/8 and AAV2/9 Vectors

**Figure 2:**
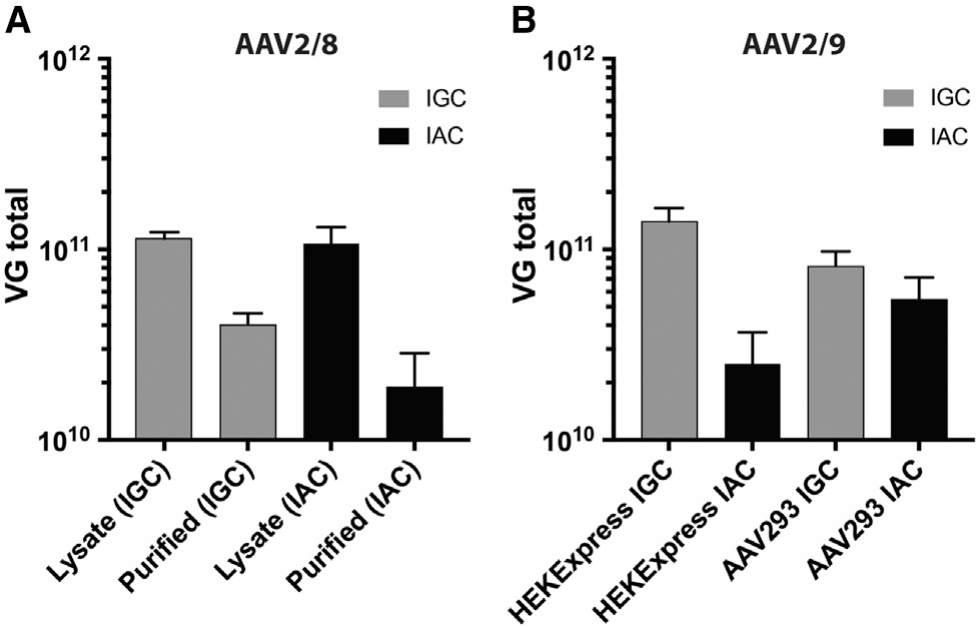
Quantification of AAV2/9 Vectors (A) Quantification of AAV2/8 viral genomes in lysates and purified vector preparations. The total yield was calculated based on the determined concentration and the fraction volume of the preparation. (B) Quantification of AAV2/9 viral genomes in IGC and IAC purified preparations deriving from HEKExpress (suspension) and AAV293 (adherent) cell lines.

The AAV2/8 (HEKExpress) and AAV2/9 (HEKExpress and AAV293) were titrated using a universal real-time PCR method for the detection of AAV sequences based on primers recognizing the ITR.^23^ The AAV8 reference material was used as an internal control. Viral genome (VG) quantification by qPCR showed that the VG content of the cleared lysates derived from two AAV2/8 batches were very similar, with 1.14 × 10^11^ and 1.07 × 10^11^ VGs (Figure 2A). A recovery of 35.6% of the initial load was obtained with the IGC (total of 4.05 × 10^10^ VGs), whereas 17.9% of vectors (total of 1.91 × 10^10^ VGs) was recovered with IAC.

Quantification of AAV2/9 vector preparation from adherent and suspension cells showed the yields of the vector preparations to be between 2.5 × 10^10^ and 1.5 × 10^11^ VGs after purification. The VG concentration in batches that were purified via IGC was higher than their corresponding batches purified via IAC; this is in agreement with the finding of the AAV2/8 purification (Figure 2B). Because the initial input was similar before purification, it is likely that this difference is due to higher loss of VG-containing particles when the vector was purified by IAC.

### *In Vitro* Analysis of AAV2/9 Vectors from Suspension and Adherent Cell Lines

**Figure 3:**
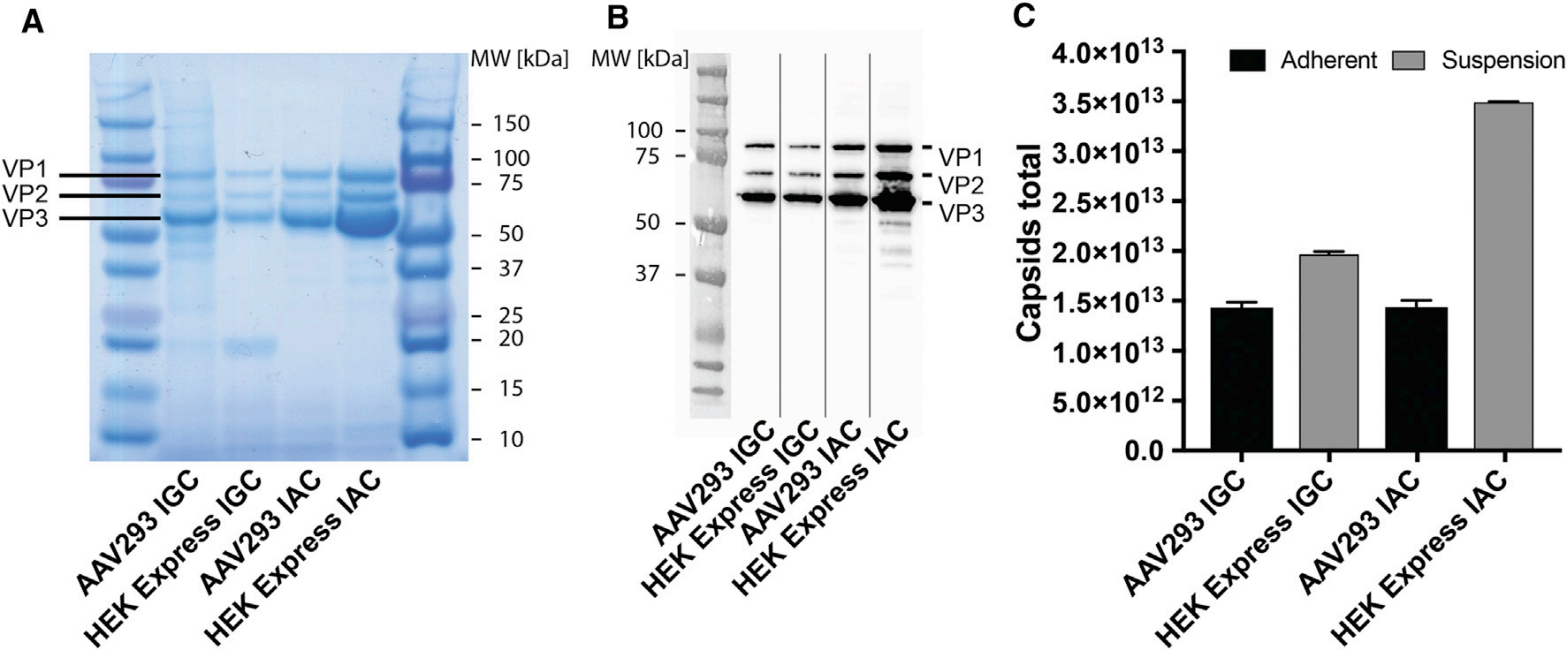
Characterization and *In Vitro* Analysis of Vector Preparations (A) The molecular weight of VP1 (87 kDa), VP2 (72 kDa), and VP3 (62 kDa) proteins is indicated. A total of 3 × 10^9^ VGs were loaded per well, and the nitrocellulose membrane was stained with Coomassie blue. (B) Western blot analysis with an anti-VP antibody B1 (5 × 10^7^ VGs per well). (C) The production of assembled capsids was analyzed by capsid ELISA analysis specific to AAV2/9. The total amount of capsid was calculated based on the determined concentration and the corresponding fraction volume.

We conducted various *in vitro* assays to characterize the AAV2/9 vector preparations in order to investigate possible differences between suspension and adherent cell lines. Initially we characterized the AAV2/9 batches by performing SDS-PAGE followed by Coomassie blue staining. The applied volumes were normalized based on VG content. Staining showed the abundant presence of VP1, VP2, and VP3 proteins in the vector preparation and confirmed the efficient removal of protein impurities from the vector batches isolated by IGC and IAC (Figure 3A). We further analyzed the presence of VP proteins by performing western blot analysis with the monoclonal VP antibody B1 (Figure 3B). The ratios between the amounts of VP1, VP2, and VP3 were similar for all four AAV2/9 batches by band-intensity measurements (Figure 3A). The VP band intensities were higher in the HEKExpress IAC batch (Figure 3B), consistent with the ELISA data (Figure 3C), given that protein loading was normalized to the VG content of the samples. This suggests that AAV2/9 produced in HEKExpress cells and purified by IAC contains a higher proportion of empty particles. We also detected protein bands from 30 to 50 kDa in the IAC-purified preparations by western blotting. Their intensity correlated with the overall amount of VP protein. These protein impurities should have been removed during ultrafiltration with a molecular weight (MW) of 100 kDa, given their size. Thus, the impurities are likely product related, because the most intense signals were found in the preparation that had the overall highest VP protein concentration and were detected with the anti-VP antibody B1 (Figure 3B, HEKExpress IAC).

We next determined the total amount of intact AAV2/9 virions by ELISA, according to the manufacturer’s protocol. The ELISA assay showed that similar quantities of assembled capsids (1.4–1.9 × 10^13^) could be obtained from either adherent or suspension cultures, using IGC as the purification method (Figure 3C). However, when the vector was purified by IAC, the number of particles produced by HEKExpress cells reached 3.4 × 10^13^. It is at this stage that the origin of the difference between total capsids and total VGs for HEKExpress cells, in particular for the IAC protocol, is unclear. We hypothesize that the high capsid concentration may have led to competition for affinity ligands on the resin, and thus ultimately to a lower VG recovery. Furthermore, we cannot exclude that, given the high capsid concentration, vector aggregation occurred after concentration.

**Figure 4:**
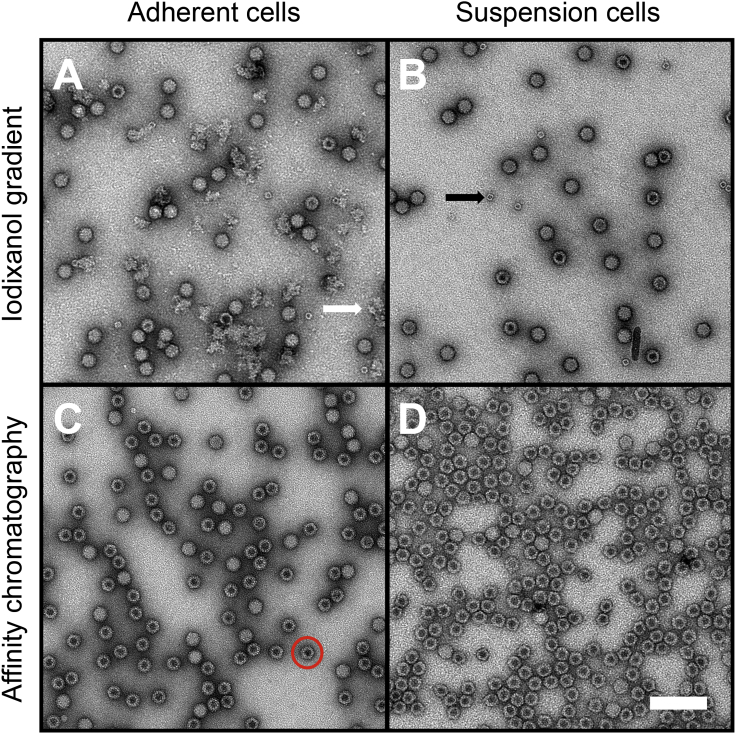
Analysis of AAV2/9 Vectors via Negative-Stain Transmission Electron Microscopy Representative images were captured at different positions on the grid. (A) AAV293 IGC particles with a full-to-empty ratio of 5.2, (B) HEKExpress IGC particles with a full-to-empty ratio of 1.4, (C) AAV293 IAC particles with a full-to-empty ratio of 0.3, and (D) HEKExpress IAC particles with a full-to-empty ratio of 0.1. One exemplary empty particle is highlighted by the red circle. Empty particles can be distinguished based on the accumulation of stain in the particle center. The black arrow indicates impurities found exclusively in preparations derived from gradient ultracentrifugation (identified by previous studies as ferritin^11^). The white arrow highlights impurities that we found only in the AAV293 IGC preparation, which also appeared to contain the highest amount of impurities for the SDS-PAGE analysis.

The production of full genome-containing particles determines the efficacy of a vector preparation. However, the presence of empty particles is also an important parameter in vector quality control. We analyzed vector preparations via negative stain transmission EM (TEM) to further determine the ratio of genome-containing to empty particles (Figure 4). Empty and full particles are clearly distinguishable by uranyl formate staining, which was used to obtain higher resolution than that obtained with uranyl acetate. We counted the particles and calculated the full-to-empty ratio. As expected, vectors purified with IGC exhibited a higher full-to-empty particle ratio (AAV293 IGC: 5.2 and HEKExpress IGC: 1.4) than preparations obtained via IAC (AAV293 IAC: 0.3 and HEKExpress IAC: 0.1). IAC does not allow the separation of full and empty vector particles, because it is based on affinity toward conformational epitopes on the surface of the capsid. In contrast, IGC is based on particle density, which allows the separation of full and empty vectors.

### *In Vivo* Analysis of Transduction Efficiency and Tropism

**Figure 5:**
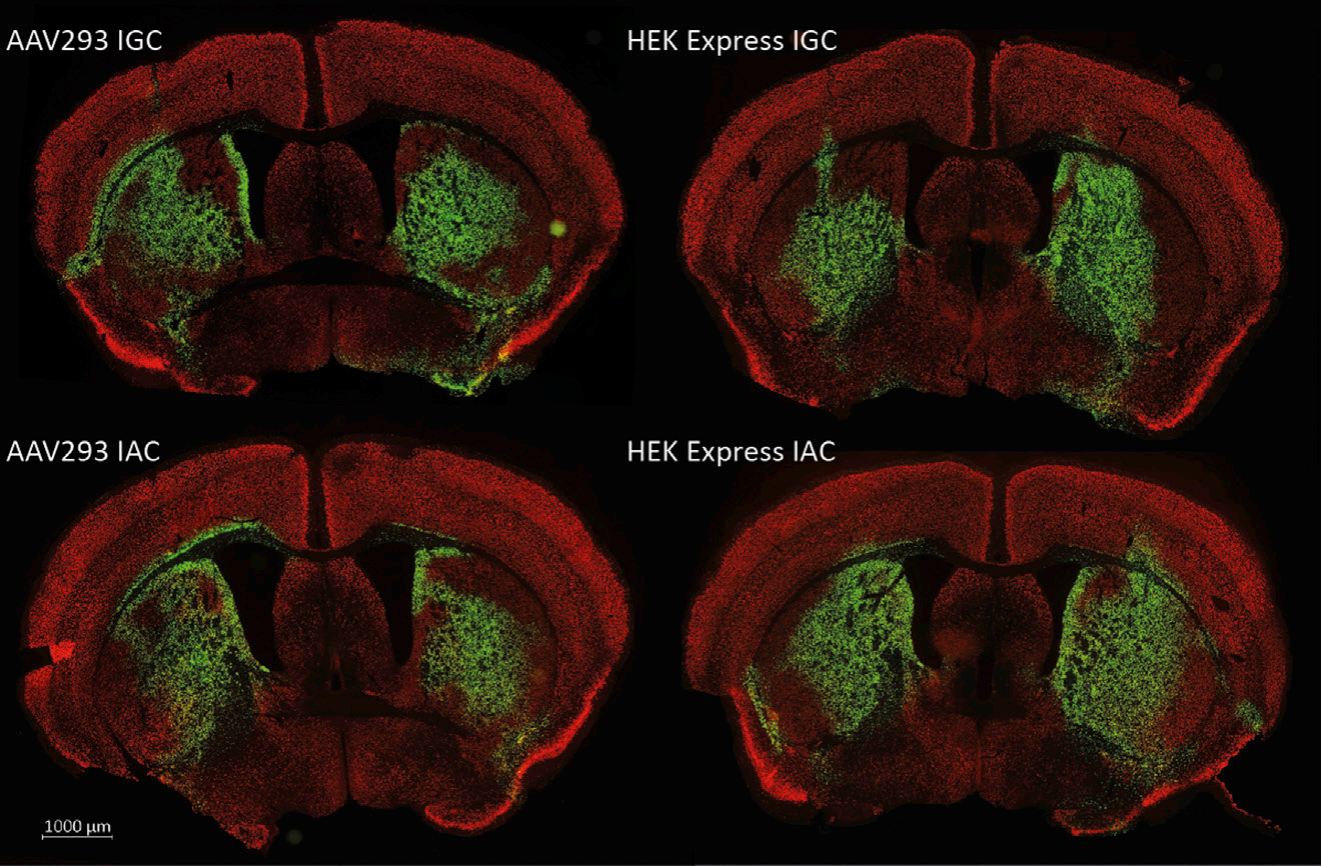
GFP Epifluorescence 4 Weeks after Injection of Respective AAV2/9 Vectors into the Striatum of 7-Week-Old C57BL/6 Mice Each vector was injected at a dose of 3 × 10^7^ VGs. Depicted sections are in proximity to the injection site. The white scale bar represents 1,000 μm.

**Figure 6:**
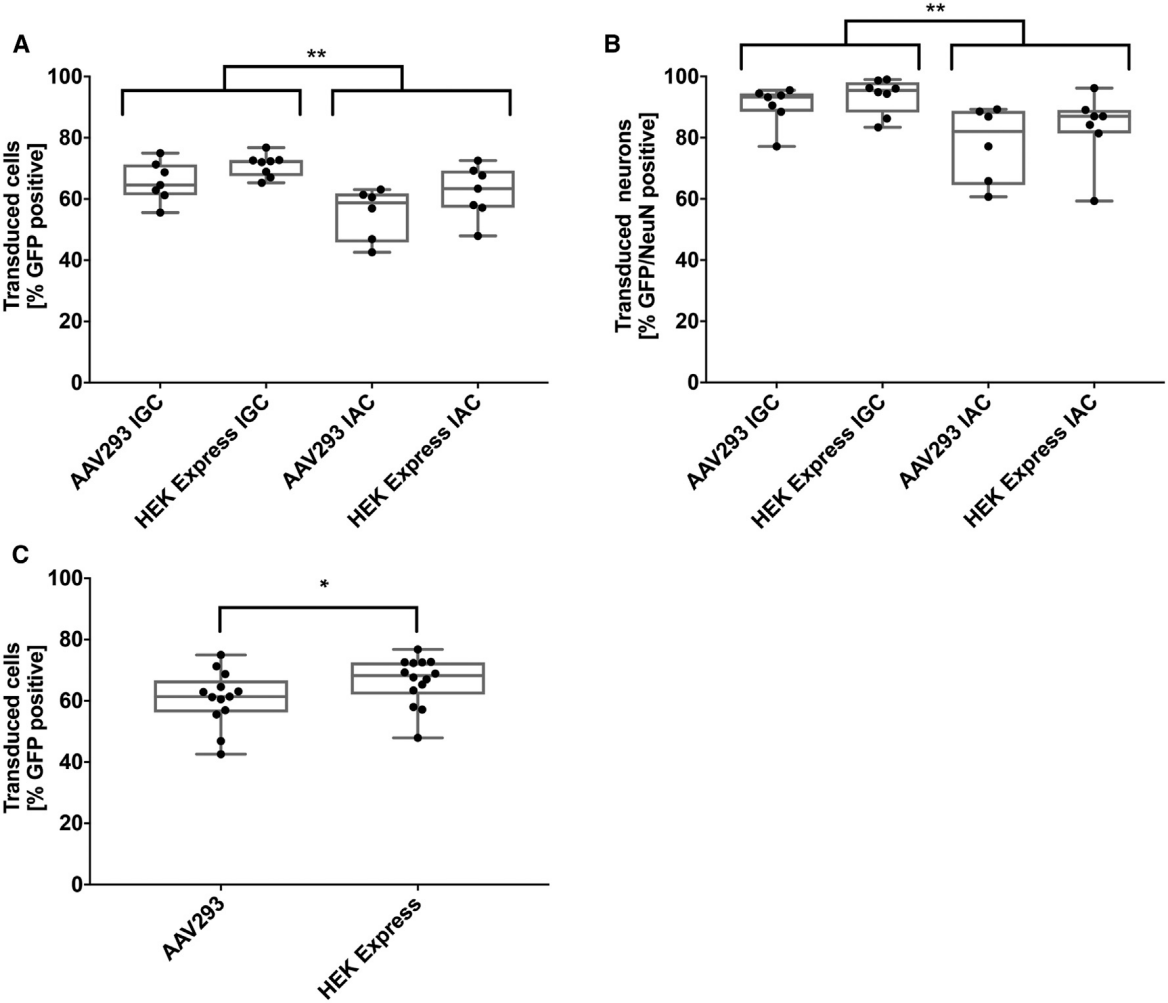
Boxplot of Transduction Efficiencies of Vector Preparations *In Vivo* (A) Quantification based on the detection of GFP-positive cells via confocal microscopy of sections close to the site of injection (two-way ANOVA, Sidak’s multiple comparison test, p = 0.0014). (B) Analysis of vector tropism based on GFP and NeuN colocalization (two-way ANOVA, Sidak’s multiple comparison test, p = 0.0037). (C) Transduction efficiency based on GFP expression, pooled according to producer cell lines (two-way ANOVA, Sidak’s multiple comparison test, p = 0.0266).

CNS gene delivery was tested by intracerebrally injecting four mice with each of the AAV2/9-CBA- nuclear localized GFP (nlsGFP) vector preparations. We bilaterally injected a total of 3 × 10^7^ VGs in a volume of 2 μL into the striatum. The vector dose was chosen to optimize the quantification and comparability of vector preparations, but not to maximize either the global level of transduction or vector diffusion. The mice were sacrificed 4 weeks after injection to assess AAV2/9-mediated transduction based on the number of GFP-positive cells. Global transduction was assessed and GFP epifluorescence measured for all sections via a microscope slide scanner. There was strong nuclear GFP expression over large portions of the striatum for all preparations (Figure 5). Preparations purified via IGC showed a statistically significantly higher median transduction efficiency than those prepared by IAC (Figure 6A). Furthermore, preparations derived from HEKExpress cells showed a higher median transduction efficiency (67.1%), regardless of the purification method (Figure 6C).

Overall, the production of cell suspension-generated AAV2/9 vectors was at least as efficient as that by adherent cell cultures with respect to transduction efficiency. We next investigated the colocalization of nlsGFP and the neuronal marker neuronbal nuclei (NeuN) to assess the tropism of the vectors. All four vector preparations displayed a similar tropism for neurons, with more than 80% of all transduced cells being NeuN-positive (Figure 6B). Furthermore, the proportion of GFP-positive neurons was consistent with the overall transduction efficiency. Two-way ANOVA revealed a statistically significant difference between groups depending on the purification method.

In conclusion, the results demonstrate that vectors deriving from suspension or adherent cell cultures exhibit similar potency and tropism when injected into the CNS of mice. Overall, these results show that transient transfection of suspension cell cultures is a scalable method for the production of AAV2/9 vectors, which can be used for effective gene delivery to the CNS.

## Discussion

Large amounts of vector are required for the validation of AAV vectors in pre-clinical animal studies and later for clinical trials. It is thus necessary to manufacture such vectors at a sufficient scale using cGMP-compliant processes to obtain the required quantity and quality. One commonly used AAV production platform for pre-clinical research is based on the plasmid-based transfection of adherent HEK293 cells grown in serum-containing media. This production method is difficult to scale up, labor intensive, and incompatible with current GMP-compliant manufacturing practices. In this comparative study, we demonstrated the production of an AAV2/9 vector using a scalable protocol for the transfection of HEKExpress cells in suspension and their purification, including a scalable IAC procedure. We characterized the vector preparations via *in vitro* analysis, providing evidence for the bioequivalence of vectors derived from adherent cells and cells in suspension, and finally verified their transduction potency in the CNS of mice.

The proof of concept for the serum-free production of AAV vectors, using HEK293 cells in suspension, in the absence of helper viruses, has been described as early as 2007.^18^, ^24^ The use of serum-free medium was a first step toward cGMP compliance, because it decreases the risk for contamination with adventitious agents.^25^ Furthermore, virus production using cells in suspension is scalable, as opposed to production with adherent cells.^26^

The use of OSRs allows the adaption and cultivation of HEK293 cells in suspension at high viabilities. OSRs are available as single-use bioreactor systems up to a scale of 1,000 L.^15^ The mixing principle of such reactors is based on horizontal movement and shaking of the entire reactor, in contrast with the mixing of stirred tank bioreactors with an impeller. Key features of OSR technology are high gas transfer rates, low mixing times, and low specific power consumption.^12^, ^13^, ^27^ These characteristics make OSR technology one of the most promising disposable bioreactor platforms for mammalian and insect cell culture for both small- and large-scale operation.^14^, ^15^, ^28^, ^29^

Previous studies have demonstrated that parameters, such as the amount of DNA, DNA-to-PEI ratio, and cell density at the time of transfection, as well as the use of butyrate or butyric acid, the medium, and the process mode in general (fed-batch, perfusion), are critical to maximize yields and decrease costs.^10^, ^11^, ^24^, ^30^, ^31^, ^32^ For our study, the total amount of DNA per cell and the DNA-to-PEI ratio for transient transfection of HEKExpress cells were based on previously optimized protocols.^33^, ^34^ Of note, this protocol reduced the amount of plasmid DNA used for PEI transfection of cells in suspension by 50% relative to that used for calcium phosphate transfection (Table 1). This is one obvious advantage of the PEI-based transfection of HEK293 cells in suspension, because highly pure plasma DNA remains one of the main cost drivers of transient production processes.

In parallel to process development, which aims to optimize upstream processing, downstream processing, particularly methods for the scalable purification of AAV vectors, has been another area of intense interest. Various methods have been explored for the purification of AAV vectors.^35^, ^36^, ^37^, ^38^, ^39^, ^40^, ^41^, ^42^ The most commonly used is iodixanol or CsCl gradient ultracentrifugation. However, methods based on discontinuous centrifugation are not feasible for large-scale manufacturing. The processing of large volumes of cell culture lysate would be extremely difficult to implement in a GMP-compliant manner. Nevertheless, these methods are frequently used and are suitable for most basic research applications and pre-clinical *in vivo* studies.^43^ Furthermore, gradient ultracentrifugation results in the very efficient separation of empty from full AAV vector particles. In contrast, affinity chromatography, although it is readily scalable and highly selective, does not discriminate between full and empty particles.^44^ By comparing IGC and IAC, we demonstrated that the removal of empty particles improved the overall transduction efficiency upon injection of vectors into the striatum. High vector concentrations are desirable for applications in the CNS, because the volume that can be delivered is limited. Thus, the removal of empty particles is a prerequisite to increase the concentration of infectious vectors and avoid the aggregation of vector particles. For applications outside of the CNS, particularly systemic delivery, the elimination of empty particles is even more important to decrease potential immunogenicity and avoid possible adverse events following the immediate immune response. Ion-exchange chromatography is the only chromatographic method that has been described for the separation of full and empty particles, based on different net charges of the respective capsids.^39^, ^45^, ^46^ In GMP processes, a minimum of two purification procedures are usually combined to achieve the required specifications in terms of product purity. Combining iodixanol density ultracentrifugation and chromatography has been successfully used for the production of clinical-grade AAV, but this strategy is difficult to scale up.^47^ As an alternative, vectors purified via affinity chromatography using the Poros CaptureSelect AAV9 resin, followed by anion exchange chromatography, have been demonstrated to yield high purity and partially remove empty particles.^45^, ^48^, ^49^

We tested the potency of the different AAV2/9 vectors by assessing cell transduction following intra-striatal injection in adult mice. The dose of AAV2/9 was not chosen to maximize diffusion and global transduction, but to facilitate the side-by-side comparison of the different AAV batches. AAV2/9, under the control of the chicken β-actin (CBA) promoter, induced high expression of the transgene, and GFP-positive cells were widely distributed throughout the injected hemisphere,^21^, ^50^, ^51^, ^52^, ^53^, ^54^ as already reported. Co-localization with the neuronal marker NeuN confirmed that the GFP-positive cells were predominantly neurons. Furthermore, the NeuN/DAPI ratio showed that neurons represented 60%–65% of striatal cells, in agreement with other studies.^55^ Our *in vivo* data show that the purification method, and thus the ratio of full to empty particles, had a limited but statistically significant effect on transduction efficiency. This finding demonstrates the feasibility of producing AAV vectors using the developed process, according to the requirements for efficient gene delivery to the CNS.

## Materials and Methods

### Plasmids

The AM/CBA-EGFP-WPRE-bGH plasmid was kindly provided by Prof. During (Auckland, New Zealand).^56^ The CBA promoter was first described by Niwa et al.^57^ The 1.7-kb chicken β-actin promoter and CMV enhancer is fused to exon 1 (90 bp) of the chicken β-actin gene, a hybrid chicken β-actin and rabbit β-globin intron (917 bp), and rabbit β-globin exon 3 (55 bp), and is also called CB or CAG in the literature. The EGFP reporter gene was removed by BamHI/EcoRV digestion and replaced by a gateway cassette (reading frame A cassette; Invitrogen) from the pBS-Gateway plasmid digested with BglII/EcoRV. Finally, a Gateway LR Clonase reaction (Invitrogen, Thermo Fisher Scientific) was performed with the resulting plasmid (pAAV2ss-CBA-Gateway-WPRE-bGH) and pENTR-AcGFP1nuc (derived from Clontech pAcGFP1-Nuc) to produce pAAV2-CBA-nlsGFP-WPRE-bGH. The pAAV-CMV-EGFP plasmid was obtained by inserting the EGFP cDNA from the pAAV-CMV-MCS plasmid (Agilent, Santa Clara, CA, USA) using standard cloning procedures. The pDP8 (Plasmid Factory, Bielefeld, Germany) or pDP9rs-gck plasmid (kindly provided by Dr. Jurgen Kleinschmidt, Heidelberg, Germany) was used for producing the AAV8 and AAV9 serotypes, respectively.

### Cell Culture

The HEKExpress cell line is the progeny of an adherently growing cell population originally called HEK293 ATCC CRL-1573, obtained from the American Type Culture Collection in 1996. This cell line was weaned off fetal bovine serum (FBS) and adapted to suspension culture over a long period (months) at the laboratory of Cellular Biotechnology, EPFL. The cells were grown in suspension culture in several different animal-component-free media, initially in Spinner Flasks (30–50 rpm) and later in OrbShake tubes (50 mL), under shaking conditions between 150 and 200 rpm (displacement radius, 50 mm). During this long-term culture, over many months, sub-cultivations were executed at shorter time intervals than typically performed. This resulted in cultures that had a very high degree of single-cell characteristics, reducing the trend of other suspension-adapted 293 cells to aggregate. In 2004, a culture of these HEK293 cells was transferred to ExcellGene (Monthey, Switzerland) and extensively cultured, eventually in EX CELL 293 medium (Sigma-Aldrich, Buchs, Switzerland), to obtain a very fast-growing and literally aggregate-free cell culture, from which a cell bank (500 vials at >20 × 10^6^ cells/vial, 2008-11-12), maintained at −80°C, was generated in 2008. This bank, now called “HEKExpress XLG1.0 MCB,” has been extensively tested using most available safety and characterization tests. It is free of human, mouse, and bovine viruses, and infection with any known adventitious agent, such as mycoplasma, bacteria, or fungi. This non-cloned cell population has a good growth rate, with a maximal cell density in batches of approximately 4–5 × 10^6^ cells/mL in EX CELL 293 medium, and a doubling time of approximately 24 hr. It has proven to be highly transfectable, with excellent expression levels for recombinant protein production. The cells can be easily transferred to and cultivated in several commercially available media that support suspension cultures.

HEK293 cells in suspension were routinely cultivated in a 50-tube TubeSpin bioreactor (TS50; TPP, Trasadingen, Switzerland) in volumes of 5–10 mL serum-free Freestyle F17 medium supplemented with 4 mM GlutaMAX (Thermo Fisher Scientific, Zug, Switzerland). Cells were orbitally shaken at 180 rpm at 37°C with 5% CO_2_ and 85% humidity in ISF-4-W incubators (Kühner, Birsfelden, Switzerland). Cells were seeded at 0.5 × 10^6^ cells/mL, independent of the cell line, and passaged twice per week by dilution into fresh medium.

Adherent HEK293 cells, called AAV293 (Agilent Technologies, Basel, Switzerland), were grown in T-flasks (25 or 75 cm^2^) in DMEM containing 10% FBS. Cells were seeded at 15%–20% confluence and were passaged twice per week before reaching 100% confluence.

### Transient Transfection of Cells in Suspension

Three to four days before the planned transfection, cells were diluted with fresh medium to a cell density of 0.5–1 × 10^6^ cells/mL in an appropriate volume for the intended scale of production (approximately a tenth of the production volume). On the day of transfection, cells were counted and diluted to a density of 1 × 10^6^ cells/mL. The transfection cocktail was prepared by adding pDNA and then PEI into F17 medium, to a total volume corresponding to 5% of the production volume. The amount of pDNA and the PEI:pDNA ratio must be optimized and depend on the vector, the transgene, and the cell line used for production. The cocktail was then mixed and incubated at room temperature for 10 min. Complex formation was verified by the increase in the turbidity of the transfection cocktail. Following incubation, the transfection cocktail was added to the cell culture, followed by the addition of CDM4HEK293 medium, corresponding to 10% of the initial cell culture volume after dilution (GE Healthcare, HyClone).

For each of the TubeSpin 600 bioreactors, a separate transfection mix was prepared in a 50-mL centrifuge tube. First, 375 μg of plasmid DNA was added to 12.5 mL of F17 medium. Equimolar amounts of the shuttle and helper plasmids were used. Subsequently, 750 μg of PEI was added to the mix, resulting in a DNA:PEI ratio of 1:2. The tubes were quickly vortexed and then incubated at room temperature for 10 min before adding the mixture to the cell cultures.

### Transient Transfection of Adherent Cells

The production with adherent cells was carried with 10 cell factories (500 cm^2^), with 5 cell factories considered as one batch. On the day before transfection, cells were seeded to a density of 4 × 10^7^ cells/cell factory in 100 mL of DMEM. A transfection mix of 10 mL was prepared for each cell factory. First, 200 μg of helper and 100 μg of shuttle plasmid were added to 2.5 mL of a 0.5 M calcium chloride solution. The mix was than adjusted to 5 mL with DNase-free water before the addition of 5 mL of a 2× HEPES buffered saline (HBS) buffer. The transfection mix was then incubated at room temperature for 10 min before adding it drop-wise to the cell factories. Six hours post-transfection the medium of the cell factories was changed and the DMEM replaced by EpiSerf (Thermo Fisher Scientific, Zug, Switzerland).

### Harvesting, Cell Lysis, and Clarification

After 72 hr (depending on the vector or transgene), the transfection was stopped, the cells counted, the viability determined, and the transfection efficiency assessed using a BD Accuri C6 (BD Biosciences, Allschwil, Switzerland) flow cytometer. The cells are then collected via centrifugation. The pellets were carefully resuspended and washed with phosphate buffer (pH 7.4). After another round of centrifugation, the supernatant was discarded and the pellet frozen at −80°C.

For further processing the cell pellet was thawed at 37°C and resuspended in phosphate buffer (pH 7.4) containing 2 mM MgCl_2_. A volume of buffer 10 times that of the packed cells was added. Cells were lysed via three freeze-thaw cycles. 3-[(3-Cholamidopropyl)dimethylammonio]-1-propanesulfonate (CHAPS) (0.5%) was then added to the lysate and the tubes mixed well and incubated at 37°C for 30 min. Following lysis, 5 U Benzonase or DNase I/million cells (cell number at the time of harvest) was added to the lysate and the tubes incubated at 37°C for 1 hr to digest the genomic DNA (observable reduction of the viscosity), residual plasmid DNA, and free VG replicates. NaCl was then added to a concentration of 150 mM to prevent aggregate formation. The lysate was initially clarified by centrifugation at 10,000 × *g* for 15 min.

### Purification via IGC

The clarified lysate was transferred to a 30-mL ultracentrifuge tube (maximal volume = 8 mL) and sequentially underlaid with 15% (9 mL), 25% (5 mL), 40% (5 mL), and 60% (3 mL) iodixanol solutions (Axon Lab, Mont-sur-Lausanne, Switzerland; PBS [pH 7.4]) The centrifugation was carried out using a 70Ti rotor at 350,000 × *g* for 90 min at 4°C. After centrifugation, iodixanol fractions were recovered by piercing the bottom and opening the top of the centrifuge tube. The first 2 mL of the 60% fraction was discarded. The next 5 mL was collected and transferred into Amicon Ultra-15, PL100 tubes (Merck, Schaffhausen, Switzerland) for buffer exchange and concentration.

### Purification via IAC

An AKTA Pure chromatography controller (GE Healthcare, Glattbrugg, Switzerland) and the POROS CaptureSelect AAV9 resin (Thermo Fisher Scientific, Zug, Switzerland) were used for IAC. Initially, an Omnifit column (Diba Industries, Danbury, CT, USA) with a diameter of 1 mm was packed according to the manufacturer’s recommendations (see POROS CaptureSelect AAV Resins: AAV8, AAV9, AAVX User Guide). The use of 10 mL of slurry and a flow rate of 10 mL/min (linear velocity ≈763 cm/hr) resulted in a packed column volume of approximately 5.6 mL. The column was equilibrated with 10 column volume (CV) of equilibration buffer (10 mM phosphate buffer [pH 7.4]) at a flow rate of 2 mL/min (linear velocity ≈152 cm/hr). The clarified lysate was loaded at a flow rate of 1 mL/min (linear velocity ≈76 cm/hr). The column was then washed with 5 CV equilibration buffer at a flow rate of 2 mL/min. A pilot study was performed to optimize the elution conditions (pH step gradient elution; data not shown). Bound AAV2/9 particles were recovered by one-step elution using 50 mM citrate buffer (pH 3) at a flow rate of 2 mL/min. Elution fractions of 5 mL were collected. Immediately after collection, neutralization buffer (100 mM Tris [pH 9]) was added to adjust the fractions to pH 7. The 280-nm absorbance peak fractions were pooled and further processed. After the elution step the column was regenerated using 5 M guanidine hydrochloride.

### Buffer Exchange and Concentration via Ultrafiltration

Amicon Ultra-50 or Ultra-15 tubes (Millipore, Zug, Switzerland) were prepared and washed according to the manufacturer’s instructions. The collected iodixanol or pH-neutralized elution fractions were initially transferred to Amicon Ultra-15 tubes (Merck, Schaffhausen, Switzerland) and centrifuged at 3,000 × *g* until the volume was reduced to approximately 500 μL. The concentrate was washed two times with 12 mL of phosphate buffer (pH 7.4), concentrated, and subsequently aliquoted for storage at −20°C.

### AAV Titration (qPCR)

The AAV ITR qPCR was based on a previously published protocol using the forward ITR primer: 5′-GGAACCCCTAGTGATGGAGTT-3′, reverse primer: 5′-CGGCCTCAGTGAGCGA-3′, and probe: 5′-FAM-CACTCCCTCTCTGCGCGCTCG-TAMRA-3′ (Microsynth, Balgach, Switzerland).^23^ The 62-bp PCR product was generated using a Rotor gene (QIAGEN, Basel, Switzerland). The PCR was performed with the Kapa probe fast qPCR master mix (Sigma-Aldrich, Buchs, Switzerland) in a 20-μL reaction volume using the following cycle parameters: 95°C for 180 s, then 40 cycles of 95°C for 3 s and 60°C for 20 s. AAV8 reference standard material was used as an internal control.^58^ This method has a limit of detection of 50 plasmid copies, which corresponds to 100 ssDNA vector genomes and 200 copies of ssAAV-ITR.^23^

### Assembled AAV2/9 Capsid ELISA

An ELISA based on a capture antibody detecting a conformational epitope (ADK9 monoclonal antibody), not present on unassembled capsid proteins, was used to quantify intact AAV9 recombinant virions (Bencard, Greifensee, Switzerland). The samples for the standard curve (lyophilized AAV9) were diluted in assay buffer (1:2 to 1:64, technical duplicates). Aliquots of the AAV2/9 batches were diluted (1:250,000 to 1:600,000) in assay buffer and incubated 1 hr at 37°C. The wells were washed three times and incubated at 37°C for 1 hr with the anti-AAV9 biotin conjugate according to the manufacturer’s protocol. After three washes, the samples were incubated with the substrate for 15 min at room temperature. The color reaction was measured with a photometer at a wavelength of 450 nm. A four-parameter logistic fit (4PL) curve was used to calculate the particle titer of the samples.

### SDS-PAGE and Western and Dot-Blot Analysis

A total of 3 × 10^9^ VGs were denatured for 10 min at 98°C in Laemmli-buffer (50 mM Tris-HCl [pH 6.8], 4% SDS, 10% glycerol, 0.1% bromophenol blue, 5% β-mercaptoethanol) and loaded on an SDS-PAGE gel (4%–12% Bis-Tris, 50 mM 3-(N-morpholino)propanesulfonic acid [MOPS], 50 mM Tris-base, 0.1% SDS, 1 mM EDTA [pH 7.7]). The electrophoresis was conducted at 120 V for 60 min. Protein bands were visualized by Coomassie blue staining (1 hr at room temperature) and de-stained in distilled water overnight.

For western blot analysis, the proteins were transferred onto a nitrocellulose membrane by wet-tank blotting in the corresponding transfer buffer (25 mM Tris-HCl, 192 mM glycine, 20% methanol, 0.03% SDS) at 50 V for 60 min. The membrane was then blocked with blocking buffer (5% milk powder in PBS) for 2 hr at room temperature.

After washing the membrane with PBST buffer (0.1% Tween), the blot was probed with primary anti-VP antibody B1 (dilution 1:20 in blocking buffer, mouse monoclonal immunoglobulin G1 [IgG1] antibody) (PROGEN, Heidelberg, Germany) and secondary horseradish-peroxidase-labeled polyclonal rabbit anti-mouse antibodies (diluted 1:1,000 in blocking buffer) (Agilent Technologies, Basel, Switzerland).

Assembled AAV2/9 capsids were evaluated by applying 1 × 10^6^ and 5 × 10^6^ VGs to a nitrocellulose membrane (soaked in transfer buffer for 5 min), placed in a microfiltration apparatus, under non-denaturing conditions. A vacuum was applied and samples pipetted onto the membrane. The wells were then washed with PBST (0.1% Tween) before the membrane was blocked with blocking buffer. For detection of assembled particles, the membrane was incubated with the AAV9-specific antibody, ADK9 (diluted 1:2 in blocking buffer, mouse monoclonal immunoglobulin A [IgA] kappa) (Progen; Bencard, Greifensee, Switzerland).

### Negative TEM Staining

A 5-μL drop of sample solution was adsorbed to a glow-discharged carbon-coated copper grid (Canemco & Marivac, Canada), washed with deionized water, and stained with 5 μL of 2% uranyl formate. The samples were imaged at room temperature using a Tecnai Spirit electron microscope equipped with an LaB6 filament operated at an acceleration voltage of 80 kV. Images were taken at magnifications from ×30,000 to ×50,000 and recorded with a 4,098 × 4,098 Eagle (FEI, the Netherlands) camera. The mean full-to-empty ratio for the various AAV2/9 batches was calculated based on the quantification of four images.

### Animals

Eight-week-old female (adult) C57BL/6 mice (Janvier, Le Genest-Saint-Isle, France) were used for the *in vivo* experiments. Mice were housed in a specific pathogen-free (SPF) facility in rat R.BTM.U × /R.ICV.6 cages (Innovive, Paris, France) and simple face Innorack racks (catalog [cat] no. RS.5.8.40), containing corn cob bedding with five mice per cage maximum. The animals were maintained in a controlled-temperature room (22°C ± 1°C) under a 14-hr light/10-hr dark cycle. Enrichments consisted of two wipes, one cardboard tunnel, and one cardboard or polysulfone house with two entrances and exits. Food (global rodent diet XP-18, vitamin-fortified, irradiated at 25 kGy; cat no. 3242; Kliba Nafag, Kaiseraugst, Switzerland) and water were provided *ad libitum*. All experimental procedures were performed in strict accordance with Swiss regulations concerning the care and use of laboratory animals (veterinary authorizations: 2782, 2888, and 3073).

### Stereotaxic Injections

Mice were anesthetized by the intraperitoneal injection of a mixture of 100 mg/kg ketamine (Ketasol; Graeub, Bern, Switzerland) and 10 mg/kg xylazine (Rompun; Bayer Health Care, Uznach, Switzerland). In brief, the head of the mouse was shaved, and the mouse was placed in the stereotaxic frame (model 963 Ultra Precise Small Animal Stereotaxic Instrument; Kopf, Tujunga, USA). During the surgery, the body temperature was controlled with a warming blanket (CMA 450 Temperature Controller; Phymep, Paris, France) and the eyes protected with 0.2% Viscotears liquid gel (Novartis, Switzerland). The scalp was disinfected with liquid povidone-iodine before incision and skull exposure. A 25G needle fixed to a syringe holder was used to pierce the skull at the appropriate coordinates. The stereotaxic coordinates of the striatum used were as follows: anteroposterior +0.5 mm from bregma, mediolateral ±2 mm and dorsoventral −3.5 mm from the skull, with the tooth bar set to align the bregma and lambda. AAV suspensions were injected with a 34-gauge blunt-tip needle (Unimed, Lausanne, Switzerland) linked to a Hamilton syringe (Hamilton Medical, Bonaduz, Switzerland) via a polyethylene catheter (Unimed, Lausanne, Switzerland). Mice received a total volume of 2 μL of AAV on either side of the head, administered at a rate of 0.2 μL/min. The needles were left in place for 5 min after the injection and were then slowly removed. The skin was sutured with 6-0 Prolene suture (B-Braun Medical, Sempach, Switzerland), and healing cream was applied to the scalp (Bepanthen Plus; Bayer, Leverkusen, Germany). Sterile 0.9% NaCl (B-Braun Medical, Sempach, Switzerland) was delivered by subcutaneous injection to prevent dehydration, and the mice were allowed to recover on a heating mat. The mice received 2 mg/mL sweetened acetaminophen (Dafalgan; UPSA, Agen, France) in water for the 3 days following the intervention to reduce pain.

Concentrated viral stocks were thawed on ice and resuspended in PBS with Pluronic F-68 (Thermo Fisher Scientific, Zug, Switzerland) by repeated pipetting before injection. For evaluation of tropism and transduction efficiency of the four AAV preparations *in vivo*, we injected 3 × 10^7^ VGs/site (2 μL at 0.2 μL/min) of the AAV2/9-CBA-nlsGFP preparations into C57BL/6 mice. Animals were sacrificed 4 weeks after the injection.

### Sample Processing and Immunostaining

The mice were sacrificed by an overdose of sodium pentobarbital (B-Braun Medical, Sempach, Switzerland) and transcardially perfused at a rate of 20 mL/min with 1× PBS for 1 min and then 4% PFA for 5 min. Brains were removed and post-fixed by incubation in 4% PFA for 24 hr at 4°C. They were then cryoprotected by incubation in 20% sucrose (Sigma-Aldrich, Buchs, Switzerland) in 1× PBS for 24 hr and then 30% sucrose in 1× PBS for 24 hr. Brains were then stored at −80°C until use. We cut 25-μm-thick coronal brain sections on a sledge microtome with a freezing stage at −30°C (Leica SM2010R; Biosystems Switzerland, Nunningen, Switzerland). Slices throughout the striatum were collected and stored in tubes, as free-floating slices in anti-freeze solution (25% glycerol [Sigma-Aldrich, Buchs, Switzerland], 30% ethylene glycol [Merck, Nottingham, UK], 25% 1× PBS, and 20% Nanopure water). Slices for direct fluorescence analysis and visualization were mounted directly on Superfrost+ microscope slides in Vectashield mounting medium for fluorescence with DAPI (Vector Labs, Burlingame, CA, USA).

For the immunofluorescence labeling of brain sections, free-floating slices were washed three times, for 10 min each, in 1× Tris-buffered saline (TBS) and blocked by incubation for 1 hr in 1× TBS supplemented with 5% normal goat serum (NGS) and 0.1% Triton X-100. Primary antibodies were diluted in 1× TBS-5% NGS-0.1% Triton X-100 and incubated overnight with the sections at 4°C. The following primary antibodies were used: rabbit polyclonal anti-NeuN antibody (1/1,000; Millipore, Zug, Switzerland), mouse monoclonal anti-S100β antibody (1:2,000; Sigma-Aldrich, Buchs, Switzerland), and rabbit polyclonal anti-GFAP antibody (1/200; Dako Schweiz, Basel, Switzerland). The following day, slices were washed three times for 10 min each with 1× TBS and incubated for 1 hr at room temperature with goat anti-rabbit IgG Alexa Fluor 594 (Invitrogen, Life Technologies, Zug, Switzerland) or goat anti-mouse IgG Alexa Fluor 594 (Invitrogen, Life Technologies, Zug, Switzerland), diluted 1:1,000 in 1× TBS-0.1% Triton X-100. Slices were then washed three times, for 10 min each, in 1× TBS and mounted on Superfrost+ slides, in Vectashield Fluorescence mounting medium with DAPI (Vector Labs, Burlingame, CA, USA).

### Images and Quantitative Analysis

Images of each brain slice were acquired with a 20× objective on a Zeiss Axio Scan Z1 (LSM software, Zeiss; Carl Zeiss Microscopy, Göttingen, Germany). The images used to quantify GFP-, NeuN-, and DAPI-positive cells for each batch of AAV were acquired with a 20× objective on a Zeiss LSM 880 with an Airyscan inverted confocal microscope (LSM software, Zeiss; Carl Zeiss Microscopy, Göttingen, Germany). We selected one section for which we acquired one picture per hemisphere in the center of the transduced area for each animal, based on the picture acquired on the Zeiss Axio Scan. Each picture was composed of four tiles with a z stack covering the whole brain section. The same acquisition parameters were used for all sections and all experimental groups. One image from the AAV293 IGC and HEKExpress IAC groups was removed from the analysis because of limited transduction.

For the quantitative analysis, the background of the images was corrected and then analyzed using an ImageJ spot detection macro, applying identical thresholds for all images. GFP-positive cells were measured using the same detection thresholds. Quantification was normalized based on a DAPI-positive cell population.

### Statistical Analysis

All data are expressed as the means ± SD. When more than two groups were compared, a two-way ANOVA with Sidak’s multiple comparison test was carried out using commercially available software (Prism 7.0; GraphPad). The statistical significance was set to a p value <0.05 for all tests.

## Author Contributions

B.L.S., N.D., and F.M.W. designed the experiments. D.B. produced the AAV batches in HEKExpress cell line and performed the immunoaffinity chromatography, *in vitro* characterizations (SDS-PAGE, western blot, and dot blot), and EM analysis. D.B. and M.R. performed the qPCR quantification and analysis. M.R. provided technical support for ELISA quantification. G.V. and C.P. performed the *in vivo* experiment. G.V. performed the immunohistology experiments, and D.B. the quantification of transduction efficiency. D.B., N.D., B.L.S., and F.M.W. wrote the manuscript, and D.B. prepared the figures. All authors revised the manuscript.

## Conflicts of Interest

F.M.W. is acting as CSO and founder of ExcellGene SA, Monthey, Switzerland. The other authors declare no conflict of interest.

## Supplemental Information

Document S1. Figure S1

Document S2. Article plus Supplemental Information
