# Supplementary material for: Scalable Production of AAV Vectors in Orbitally Shaken HEK293 Cells
Source: Mol Ther Methods Clin Dev. 2018 Nov 22;13:14–26. doi: 10.1016/j.omtm.2018.11.004 (PMC6305802; doi:10.1016/j.omtm.2018.11.004)
Supplement: Document S1. Figure S1 [file mmc1.pdf]

OMTM, Volume *13*

Supplemental Information

Scalable Production of AAV Vectors in Orbitally Shaken HEK293 Cells

Daniel Blessing, Gabriel Vachey, Catherine Pythoud, Maria Rey, Vivianne Padrun, Florian M. Wurm, Bernard L. Schneider, and Nicole Déglon

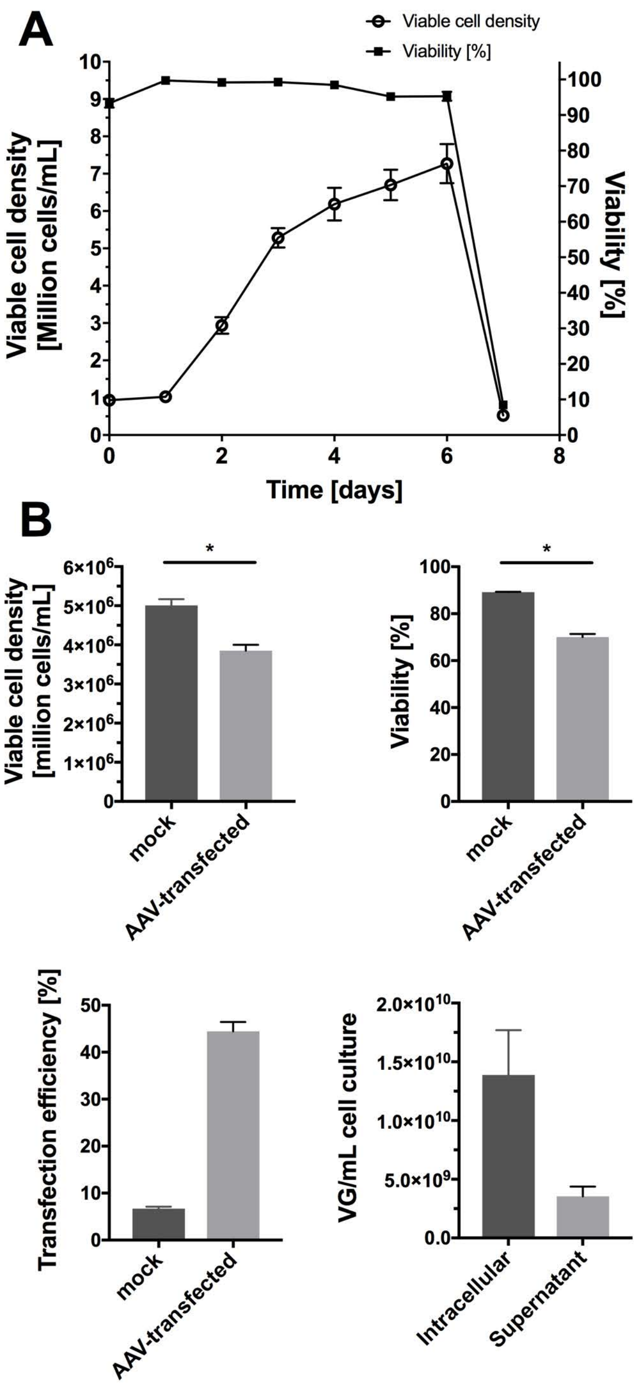

A
Viable cell density
Viability [%]
Viable cell density [Million cells/mL]
Viability [%]
Time [days]
B
Viable cell density [million cells/mL]
mock
AAV-transfected
Viability [%]
mock
AAV-transfected
Transfection efficiency [%]
mock
AAV-transfected
VG/mL cell culture
Intracellular
Supernatant
